# 2-phenylethynesulfonamide inhibits growth of oral squamous cell carcinoma cells by blocking the function of heat shock protein 70

**DOI:** 10.1042/BSR20200079

**Published:** 2020-03-12

**Authors:** Liang Jiang, Jing Xiao

**Affiliations:** 1 Department of Oral and Maxillofacial Surgery, Tongji Hospital of Tongji Medical College, Huazhong University of Science and Technology, No. 1095 Jiefang Avenue, Qiaokou District, Wuhan City, 433030, China; 2 Department of Anesthesiology, Tongji Hospital of Tongji Medical College, Huazhong University of Science and Technology, No. 1095 Jiefang Avenue, Qiaokou District, Wuhan City, 433030, China

**Keywords:** Hsp70, OSCC, PES, XIAP

## Abstract

Oral squamous cell carcinoma (OSCC) is the most common malignancy in the oral cavity, which accounts for >90% of all diagnosed oral cancers. 2-phenylethynesulfonamide (PES) was known as a selective heat shock protein 70 (Hsp70) function inhibitor, which induced cytotoxic effects on various tumor cell types, but showed to be less toxic to normal cells. However, no associated evaluation of PES on OSCC was found. In the present study, the proliferation of OSCC cells treated with PES was analyzed using a CCK-8 assay. The effects of PES on the cell cycle and apoptosis of OSCC cells were determined by flow cytometric analyses. Expression of associated protein was determined by Western blot analysis. The results of the present study showed that PES inhibited the proliferation of OSCC cell lines *in vivo* and *in vitro*. PES induced apoptosis and arrested the cell cycle of OSCC cells. PES inhibited the expression of X-linked inhibitor of apoptosis protein (XIAP), baculoviral IAP repeat containing 2 (c-IAP1), phosphorylated AKT (p-AKT), and phosphorylated extracellular signal-regulated kinase (p-ERK). Additionally, knockdown of Hsp70 enhanced the effects of PES. By contrast, overexpression of Hsp70 attenuated the inhibitory effects of PES on cell viability. PES disrupted the interaction between Hsp70 and XIAP. In conclusion, the present study demonstrated that PES suppresses the growth of OSCC cells through Hsp70-dependent mechanism.

## Introduction

It is estimated that there were ∼34000 new cases of oral cancer and 7000 oral cancer-associated deaths in the United States in 2018 [1]. Oral squamous cell carcinoma (OSCC) is the most common oral cavity malignancy, which accounts for >90% of all diagnosed oral cancers [2]. Although the OSCC 5-year survival rate has increased from 56 to 62% due to improvements in surgical and chemotherapeutic techniques [3], development of new therapeutic strategies is still important.

Heat shock proteins (Hsps) family is a group of highly preserved proteins which efficiently facilitate proper protein folding, repair denatured proteins, and prevent accumulation of inaccurate folded proteins, thereby protecting normal cells against different harmful stimulation [4]. Hsp70 binds to false folded polypeptides, re-folds these client proteins, and then transmits them to Hsp90 [4,5]. In this way, Hsp70 accelerates protein transports or post-translational modifications, promotes degradation of improperly folded substrates, and eventually maintains intracellular homeostasis [6,7]. Hsp70 has been reported to be associated with various malignancies [8,9]. Several studies have found that HSP70 expression was significantly higher in OSCC cells than normal epithelium specimens, which suggested that Hsp70 may be a potential therapeutic target for OSCC [10,11].

2-phenylethynesulfonamide (PES), also named pifthrin-μ, is known as a selective Hsp70 function inhibitor, which induces tumor cell death but markedly shows less toxicity to normal cells [12]. Mechanically, PES interacts with the substrate-binding domain of Hsp70 carboxyterminal and disrupts association between co-chaperones and substrate proteins of Hsp70 [12,13]. PES induces death of cancer cell through accumulating misfolded proteins, impairing processes of autophagy, and inhibiting functions of lysosome [14,15].

In the present study, PES suppressed proliferation, induced apoptosis, and caused the cell cycle arrest of OSCC cells. PES down-regulated expression of X-linked inhibitor of apoptosis protein (XIAP), baculoviral IAP repeat containing 2 (c-IAP1), phosphorylated extracellular signal-regulated kinase (p-ERK) and phosphorylated AKT (p-AKT) in OSCC cells. Knockdown of Hsp70 enhanced the effects of PES. Additionally, PES disturbed the interaction between Hsp70 and XIAP in OSCC cells. Furthermore, PES suppressed the growth of SCC25-induced xenograft tumors *in vivo*.

## Materials and methods

### Data acquisition and processing

Data of dependent cell lines in the Cancer Cell Line Encyclopedia (CCLE) was obtained from Depmap website (https://depmap.org/portal/) [16]. Dependence score < −0.5 was considered to indicate a statistical significance. A lower score means that a gene is more likely to be dependent in a given cell line.

### Cell lines and reagents

SCC25 and CAL27 cells were kindly provided by Dr. Zilong Gao (University of Wuhan, Wuhan, China). All cell lines were cultured in RPMI-1640 media (HyClone; GE Healthcare Life Sciences) supplemented with 10% FBS (Gibco; Thermo Fisher Scientific, Inc.) in a humidified atmosphere of 5% CO_2_ and 37°C. PES (Merck Millipore, MA, U.S.A.) was dissolved in dimethylsulfoxide (DMSO; Sigma–Aldrich, St. Louis, MO, U.S.A.) as a stock solution, and diluted to a working concentration with PBS prior to use.

### Plasmid

The plasmid pEF-Hsp70-Flag was a gift from Jingchao Wang (University of Wuhan) [9].

### Cell viability assay

SCC25 and CAL27 cells (1 × 10^4^ cells/well) were seeded in 96-well plates and treated with DMSO control (0.01%) or increasing concentrations of PES (5, 10, 20, and 40 μM) at 37°C. After incubation for 24, 48, and 72 h, cells were treated with 10 μl Cell Counting Kit-8 reagent (Dojindo Molecular Technologies, Inc.) and the plates were incubated at 37°C for 1 h in dark. The optical density (OD) was measured at an absorbance of 450 nm using an ELx800 microimmunoanalyser (BioTek Instruments, Inc.).

### Colony-formation assay

SCC25 and CAL27 cells were placed in 35-mm culture dishes (500 cells/dish) and cultured in standard medium with DMSO control (0.01%) or PES (5 μM) for 2 weeks. Units of colony formation were stained with 0.5% (w/v) Crystal Violet prepared in 0.6% (v/v) glutaraldehyde solution for 1 min. Cell colony in culture dishes were photographed.

### Cell cycle analysis

SCC25 and CAL27 cells were seeded in six-well plates and treated with DMSO (0.01%) or PES (60 μM) at 37°C for 24 h. Cells were digested and washed twice with cold PBS solution, and then resuspended in cold 75% ethanol. After fixation at −20°C for 24 h, cells were collected and resuspended in 0.5 ml cold PBS. Cells were mixed with reagent A [Multisciences (Lianke) Biotech Co., Ltd.] and incubated at 4°C for 30 min in darkness. Cell cycle analysis was performed using a Beckman Coulter system (EPICS Altra II; Beckman Coulter, Inc.).

### Cell apoptosis analysis

Cells were digested after treating with PES (20 and 40 μM) at 37°C for 24 h. Apoptosis of OSCC cells was detected using Annexin V-FITC/propidium iodide (PI) apoptosis detection kit [Multisciences (Lianke) Biotech Co., Ltd.] as described by the manufacturer. Cell apoptosis as immediately analyzed using a Beckman Coulter system (EPICS Altra II; Beckman Coulter, Inc.).

### Western blot analysis

At 4 h post-transfection, cells (SCC25 and CAL27) were washed with PBS and treated with DMSO (0.01%) or PES (10, 20, and 40 μM). After 48 h of incubation, cells were harvested and dissolved in RIPA lysis buffer (Beyotime Institute of Biotechnology) with 0.5% cocktail protease inhibitor (Roche Diagnostics). Following incubation on ice for 10 min, cell lysates were collected and sonicated for 20 s. Protein concentration was determined using a BCA assay (Bio-Rad Laboratories, Inc.). Quantified proteins were mixed with 5× loading buffer [250 nM Tris/HCl (pH 6.8), 0.5% Bromophenol Blue, 50% glycerol, 10% SDS, 5% β-mercaptoethanol] and boiled for 5 min. Lysates were separated by SDS/PAGE on 10% gels, then subjected to immunoblot analyses. The primary antibodies used were as follows: glyceraldehyde-3-phosphate dehydrogenase (GAPDH, cat no. 10494-1-AP; 1:5000; ProteinTech Group, Inc.); caspase-3 (cat no. 9662; 1:1000; Cell Signaling Technology, Inc.); cleaved caspase-9 polyclonal antibody (c-caspase 9; cat no. 20750; 1:1000; Cell Signaling Technology, Inc.); poly (ADP-ribose) polymerase (PARP-1; cat. no. 9542; 1:1000; Cell Signaling Technology, Inc.); p-ERK (cat no. 4370; 1:1000; Cell Signaling Technology, Inc.); p-AKT (cat no. 4060; 1:1000; Cell Signaling Technology, Inc.); XIAP (cat no. 14334; 1:1000; Cell Signaling Technology, Inc.); c-IAP1 (cat no. 7065; 1:1000; Cell Signaling Technology, Inc.); Hsp70 (cat no. 46477; 1:1000; Cell Signaling Technology, Inc.); anti-Flag (cat no. CFLKT002; 1:1000; Beijing Chunfenglv Biomedical Technology, Inc.).

### Cell transfection

An si-RNA targeting Hsp70 (Ribobio, Co., Ltd.) or a non-specific control were transfected to cells using X-treme GENE HP DNA Transfection Reagent (Roche Diagnostics) according to the manufacturer’s instructions. pEF-Hsp70-Flag or pEF(vehicle control) was transfected to cells using X-treme GENE HP DNA Transfection Reagent. The siRNA primers of Hsp70 were as follows: forward, 5′-AGAAGAAGGUGCUGGACAAdTdT-3′ and reverse, 5′-dTdTUCUUCUUCCACGACCUGUU-5′.

### Animal studies

The Tongji Medical College Committee on the Use and Care of Animals approved the animal assay in the present study. (H201830) A total of 10 BALB/c nude mice were obtained from the Beijing HFK Bioscience Co. Ltd. (Beijing, China) and housed in a specific pathogen-free environment. The mice experiment was performed in Laboratory Animal Center of Tongji Medical College. After 6–7 weeks old, the mice’s skin surface were anesthetized with 2% cocaine hydrochloride. Then, SCC25 cells (5 × 10^6^/100 µl) were subcutaneously injected into the flanks. When the tumor became visible (∼7 days), mice were randomly divided into two groups (*n*=5/group). The control group mice were injected intraperitoneally with PBS (0.01% DMSO) and PES group were injected with PES (20 mg/kg, three times a week). The mice were observed every day. Mice were killed after 21 days of treatment by cervical dislocation. After breathing and heart stopped completely, weight of mice and tumor sizes were measured. Tumor volume was calculated as 0.5 × length × width^2^. Tissue samples were fixed in 10% neutral buffered formalin, embedded and sectioned (3-µm-thickness).

### Co-immunoprecipitation assays

SCC25 cells (5 × 10^6^) were seeded in 10-cm dishes and transfected with plasmids for 48 h. The cells were harvested using lysis buffer (50 mM Tris/HCl, pH 7.6, 150 mM NaCl, 10 mM NaF, 2 mM EGTA, 1 mM Na_3_VO_4_, 0.5%Triton-X-100, and 2 mM DTT) for 30 min on ice. After centrifuging at 12000×***g*** for 20 min at 4°C, the supernatants were collected and incubated with the Protein A/G PLUS-Agarose beads with appropriate primary antibody or IgG isotype control antibody at 4°C overnight. The complexes were mixed with 2× loading buffer and boiled for 5 min at 100°C after washing four times. Then the immune compounds were subjected to Western blotting.

### Immunohistochemistry analysis

Immunohistochemistry was performed using antibodies against Hsp70 (cat no. 46477; 1:1000; Cell Signaling Technology, Inc.) and cleaved caspase-3 (cat. no. 9664; 1:1000; Cell Signaling Technology, Inc.) according to the manufacturer’s instructions. After deparaffinization, the tissue sections were boiled in citric acid (pH 6.0) for 20 min and immersed in 3% H_2_O_2_ for 10 min for quenching the activity of endogenous peroxidase. After blocking in goat serum for 1 h at 20–28°C, the tissue sections were incubated with primary antibodies overnight at 4°C. After washing, the tissue sections were incubated with secondary antibody (MaxVision™ Kits; MaxVision Biosciences, Inc.) conjugated to horseradish peroxidase. Then, tissue sections ere incubated with diaminobenzidine for 1 min and lightly counterstained with Hematoxylin.

### Statistical analysis

Data are presented as the mean ± standard deviation of three independent experiments. For the comparison of two groups, Student’s *t* test was selected. For the comparison of multiple groups, one way ANOVA followed by Newman–Keuls post hoc test was carried out. All statistical analysis was analyzed by using GraphPad Prism for Windows version 5.0 (GraphPad Software, Inc.). *P*<0.05 was considered to indicate a statistically significant difference.

## Results

### Growth of OSCC cell lines depends on expression of Hsp70

**Figure 1:**
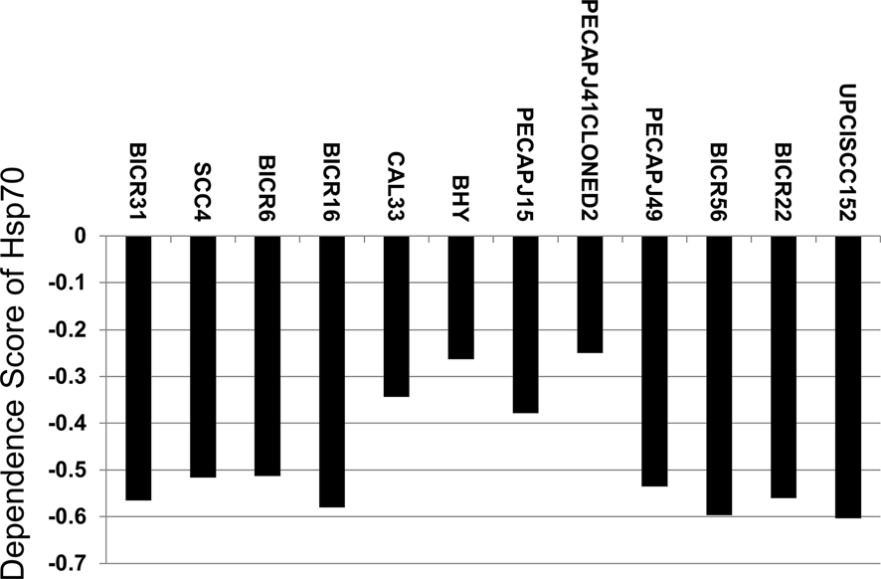
Hsp70 is a dependent gene of OSCC cells Dependence score of Hsp70 knockdown on 12 OSCC cell lines reflecting degree of gene dependence were collected from CCLE database.

To explore the effect of Hsp70 expression on growth of OSCC cell lines, the CCLE database was searched. As shown in Figure 1, among the 12 included OSCC cell lines (BICR31, SCC4, BICR6, BICR16, CAL33, BHY, PEAPJ15, PEAPJ41CLONED2, PEAPJ49, BICR56, BICR22, UPCISCC152), the dependence score of 8 OSCC cell lines (BICR31, SCC4, BICR6, BICR16, PEAPJ49, BICR56, BICR22, UPCISCC152) showed statistical significance when inhibiting Hsp70 expression by using Achilles CRISPR system (value < −0.5). These results suggested that Hsp70 may be a dependent gene of OSCC cells.

### PES effectively suppresses proliferation of OSCC cells

**Figure 2:**
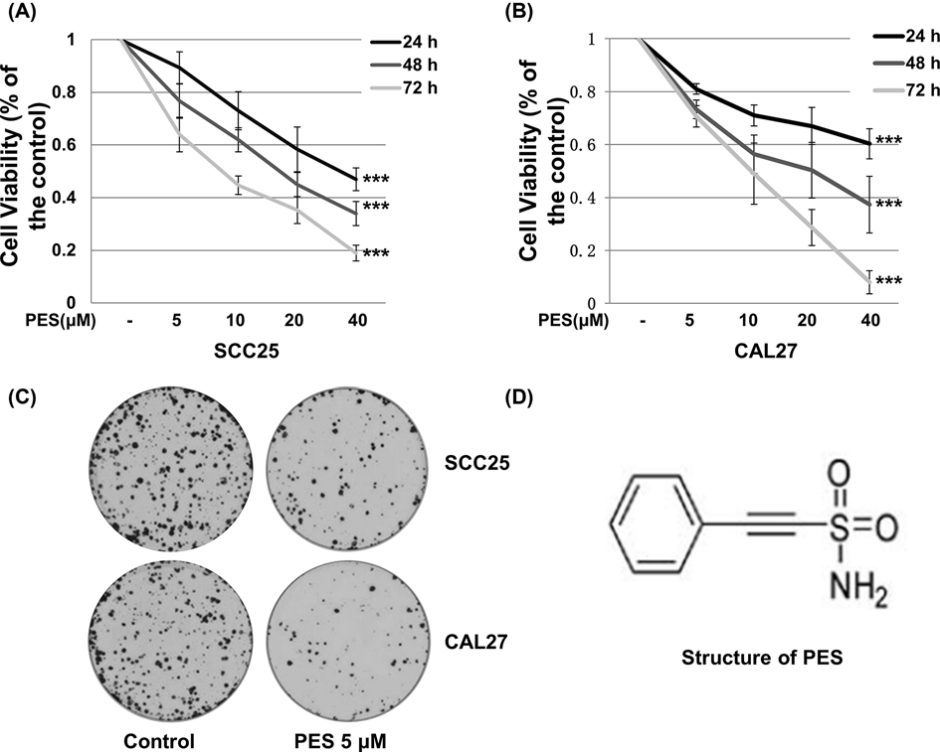
PES suppresses the proliferation of OSCC cells (**A**) SCC25 and (**B**) CAL27 were treated with 0.01% DMSO or a series of increasing PES concentrations (5, 10, 20, and 40 μM) for 24, 48, and 72 h, with cell viability was detected using a CCK-8 assay. (**C**) SCC25 and CAL27 were treated with 0.01% DMSO or PES (5 μM) for 2 weeks. Cells were stained with Crystal Violet. (**D**) Chemical structure of PES. All of the experiments were repeated three times. The values shown are mean ± SD of three independent experiments carried out in triplicate. One-way ANOVA test was used to compare significance between experiment groups and control group. ***P<0.001.

To determine whether PES decreases the viability of OSCC cells, SCC25 and CAL27 cells were treated with various doses of PES and for different durations. As shown in Figure 2A,B, PES significantly decreased the viability of SCC25 and CAL27 cells compared with cells treated with DMSO control. To further confirm the above results, colony formation assay was performed. SCC25 and CAL27 cells were placed in 35-mm culture dishes, and treated with DMSO control or PES for 2 weeks, and stained with Crystal Violet. As shown in Figure 2C, low concentration of PES remarkably reduced colony formation at both colony numbers and size. The chemical structure of PES is shown in Figure 2D. These results suggest that PES effectively inhibits the proliferation of different OSCC cell lines.

### PES induces apoptosis in OSCC cells

**Figure 3:**
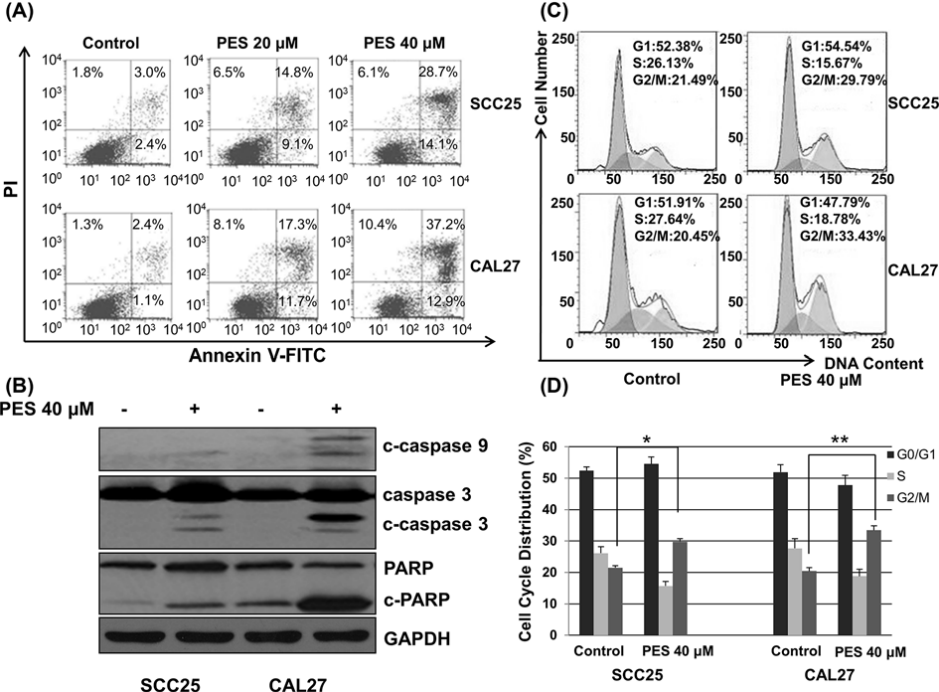
PES induces apoptosis and stops the cell cycle of OSCC cells (**A**) SCC25 and CAL27 cells were treated with 0.01% DMSO or PES (20 and 40 μM) for 24 h. Apoptosis analysis was determined by flow cytometry. (**B**) Expression of apoptosis-associated proteins (c-caspase 9, caspase 3, c-caspase 3, PARP, c-PARP) was detected by Western blotting analysis. (**C**) SCC25 and CAL27 cells were treated with 0.01% DMSO or PES (40 μM) for 24 h. Cell cycle analysis was performed by flow cytometry. (**D**) Cell cycle distribution was quantified. All the experiments were repeated three times. *P<0.05. and **P<0.05.

To examine the apoptotic effect induced by PES in OSCC cells, SCC25 and CAL27 cells were treated with increasing doses of PES for 24 h. As shown in Figure 3A, PES significantly induced apoptosis of SCC25 and CAL27 cells. To further evaluate stimulation of the apoptotic pathway, expression of several apoptosis-associated proteins was detected by Western blot analysis. PES increased the protein levels of cleaved caspase-9, cleaved caspase-3, and cleaved PARP, and decreased expression of full-length PARP and caspase-3 in SCC25 and CAL27 cells (Figure 3B). These results suggested that PES induced OSCC cell apoptosis via the mitochondrial apoptotic pathway.

### PES arrests the cell cycle in G_2_/M phase

To determine whether PES affects the cell cycle, SCC25 and CAL27 cells were treated with DMSO control or PES (40 μM) for 24 h. The distribution of cells in different cell cycle phases was analyzed by flow cytometry. As shown in Figure 3C,D, PES treatment retarded cell cycling with a reduction in S phase. The fraction of SCC25 and CAL27 cells in S phase was decreased by 10.46 and 8.86%, respectively, when treated with PES (40 μM). The G_2_/M phase was increased by 8.79 and 13.02% in SCC25 and CAL27 cells, respectively. These results demonstrated that PES reduces the proliferation of OSCC cells by arresting the cell cycle in G_2_/M phase.

### PES decreases expression of XIAP, c-IAP1, p-AKT, and p-ERK

**Figure 4:**
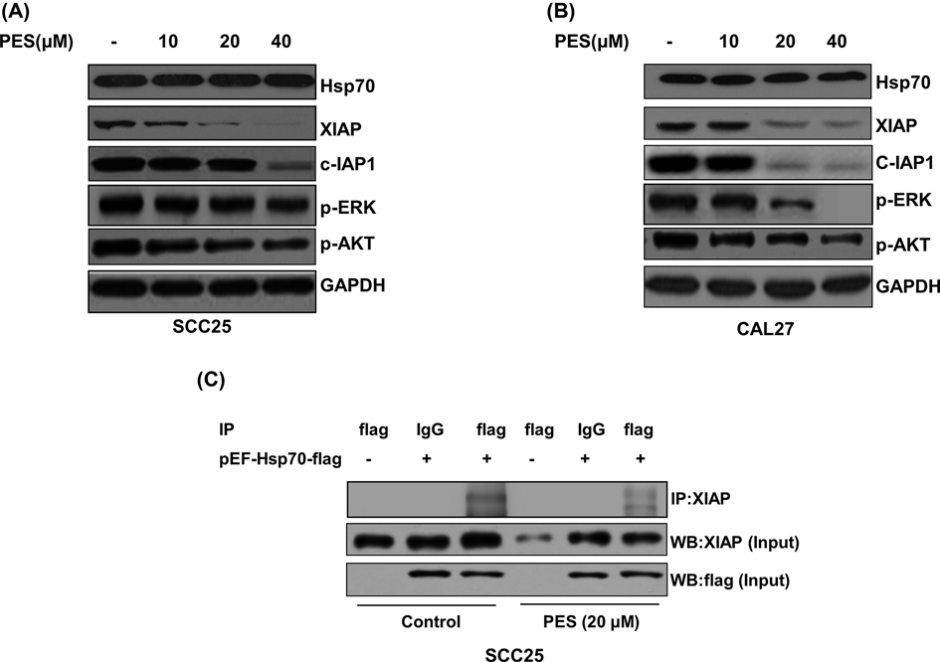
PES inhibits the biological function of Hsp70 in OSCC cell lines (**A**) SCC25 and (**B**) CAL27 cells were treated with 0.01% DMSO or PES (10, 20, and 40 μM) for 48 h. The expression of Hsp70, XIAP, c-IAP1, p-ERK, and p-AKT was measured by immunoblot analysis as indicated. (**C**) SCC25 cells were transfected with pEF-Hsp70-flag or control vector, followed by treatment with the DMSO control or PES (20 μM) beginning at 4 h after transfection. IP assays were performed after 48 h treatment. All the experiments were repeated three times. The values shown are mean ± SD of three independent experiments carried out in triplicate.

To determine whether PES works on the function of Hsp70 in OSCC cells, SCC25 and CAL27 cells were treated with DMSO control or PES. As shown in Figure 4A,B, PES did not decrease Hsp70 expression in SCC25 and CAL27 cells. XIAP and c-IAP1 were client protein of Hsp70 [17,18]. Therefore, the expression levels of XIAP and c-IAP1 were detected. The levels of XIAP and c-IAP1 were decreased by treatment with PES. In addition, PES significantly reduced expression of p-AKT and p-ERK. These results suggested that PES might inhibit the function of Hsp70 in OSCC cells.

### PES disrupts interaction of Hsp70 and XIAP in OSCC cells

XIAP was reported to interact with Hsp70 [17]. To determine whether PES works on Hsp70 in OSCC cells, a co-immunprecipitation assay was performed. As shown in Figure 4C, PES significantly disrupted the association between Hsp70 and XIAP, which suggested that PES inhibits the function of Hsp70 in OSCC cells.

### PES inhibits growth of OSCC cells through Hsp70-dependent method

**Figure 5:**
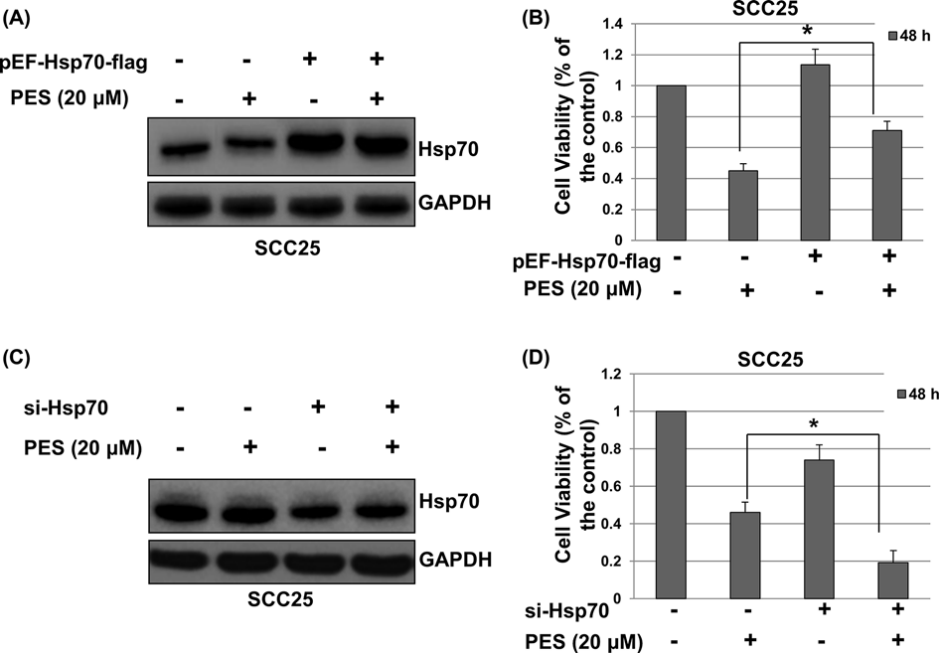
PES decreases proliferation of OSCC cell lines through inhibiting Hsp70 (**A**) SCC25 were transfected with pEF-Hsp70-flag or control vector for 4 h, and treated with 0.01% DMSO or PES (20 μM) for 48 h. The expression of Hsp70 was measured by immunoblot analysis. (**B**) Cell viability were determined by CCK-8 assay. (**C**) SCC25 were transfected with si-Hsp70 or control siRNA for 4 h, and treated with 0.01% DMSO or PES (20 μM) for 48 h. The expression of Hsp70 was measured by immunoblot analysis. (**D**) Cell viability were determined by CCK-8 assay. All the experiments were repeated three times. The values shown are mean ± SD of three independent experiments carried out in triplicate. Student’s *t* test was used to compare significance between two groups. *P<0.05.

To determine whether the decrease in cell viability was associated with the expression of Hsp70 in OSCC cells, SCC25 cells were transfected with the pEF-Hsp70-flag plasmid or siRNA-Hsp70. Total proteins were harvested and subjected to Western blot analysis. As shown in Figure 5A–C, PES treatment did not affect expression of overexpression or knockdown of Hsp70. Cell viability was determined by CCK-8 assay. As shown in Figure 5B, overexpression of Hsp70 significantly attenuated the cell killing effect of PES (*P*<0.05). As shown in Figure 5D, knockdown of Hsp70 by siRNA significantly contribute to the inhibition effect of PES on cell viability. These results suggest that PES inhibits cell proliferation of OSCC cells through Hsp70-dependent manner.

### *In vivo* effects of PES on BALB/c mice inoculated with SCC25 cells

**Figure 6:**
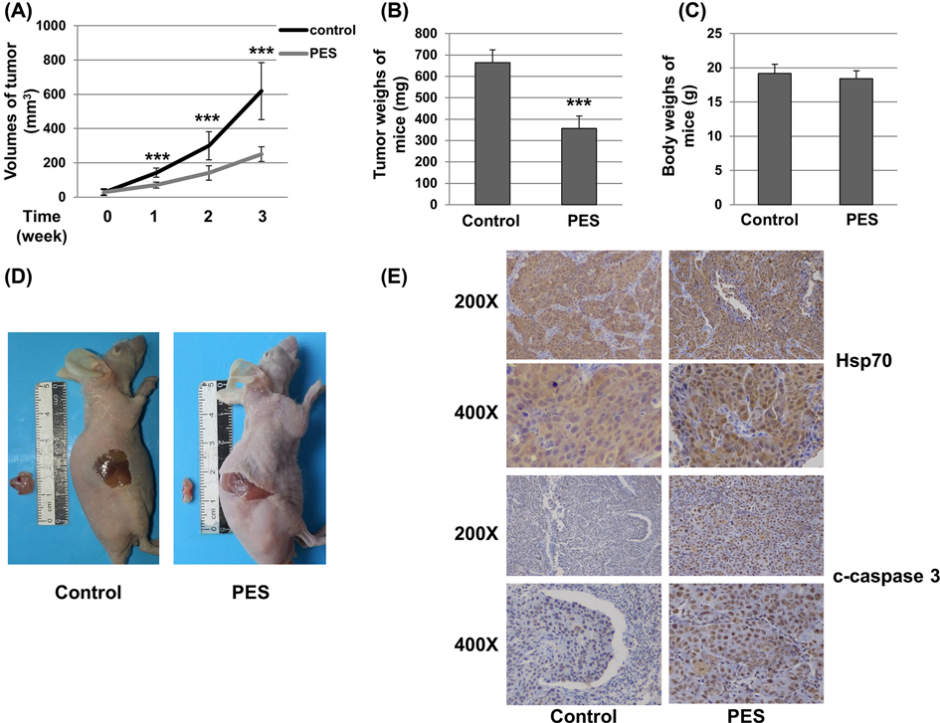
PES inhibits the growth of OSCC cell-induced tumors *in vivo* BALB/c nude mice with SCC25 cell-induced tumors were treated with DMSO (0.01%) or 20 mg/kg/day PES (*n*=5 per group) for 21 days. (**A**) Tumor volumes, (**B**) tumor weights, and (**C**) mice weights were evaluated. (**D**) Representative photos of the dissected tumors and mice were shown. (**E**) The expression of Hsp70 and cleaved caspase-3 in tumor tissues was determined by immunohistochemical analysis. Student’s *t* test was used to compare significance between two groups. ***P<0.001.

To determine the effects of PES on OSCC *in vivo*, SCC25 cells were subcutaneously inoculated into the armpit of BALB/c nude mice. The mice were treated with PES or DMSO at 7 days after tumor cell inoculation. After treatment with PES or DMSO for 21 days, mice were killed. The weights of mice were also evaluated. The weights and volumes of tumors were measured. Tumors volumes (Figure 6A) and weights (Figure 6B) were decreased by treatment with PES. Additionally, there was no significant difference in the weight of the mice between the two groups (Figure 6C). Representative images of tumors were given in Figure 6D. The expression and distribution of Hsp70 and cleaved caspase-3 were determined by immunohistochemical analysis of the tumor tissues. As shown in Figure 6E, Hsp70 expression showed no significant difference between control group and PES group. Conversely, cleaved caspase-3 expression was stronger in the PES-treated group than in the control group. These results suggest that PES decreases the proliferation of OSCC cell xenografts *in vivo*.

## Discussion

Increasing evidence indicates that Hsp70 may be a novel therapeutic target for cancer treatment, as Hsp70 is involved in cancer growth, metastasis, and development [19]. In the present study, PES, a specific Hsp70 inhibitor, effectively suppressed the proliferation of OSCC cell lines. PES induced apoptosis of OSCC cells via the mitochondrial apoptotic pathway, and arrested the cell cycle in the G_2_/M phase. PES significantly decreased the expression of XIAP, c-IAP1, p-AKT, and p-ERK. Additionally, knockdown of Hsp70 enhanced the effects of PES. By contrast, overexpression of Hsp70 attenuated the inhibitory effects of PES on cell viability. PES disrupted the interaction between Hsp70 and XIAP. Furthermore, PES decreased the growth of SCC25-induced tumor xenografts *in vivo*.

PES, the biologic function of a small molecular Hsp70 inhibitor, is found to be effective against various cancers [9,20,21]. Hsp70 is overexpressed in various malignances, including OSCC [8–11]. According to the results of Achilles CRISPR in CCLE database, knockdown Hsp70 significantly stops growth of most OSCC cell lines. In the context of this, the effect of PES against OSCC was investigated. PES is found to induce apoptosis via both the intrinsic and extrinsic apoptosis pathways in cancer cells [20,22]. For example, PES treatment induces caspase-3 and caspase-9 activation, eventually activing expression of cleaved PARP in lung cancer [20]. PES also induces caspase-8-dependent death receptor-mediated apoptotic pathways in bladder cancers [22]. Aside from pro-apoptosis effects, PES also regulates the cell cycle. By inhibiting expression of cyclin A and CDK2, PES arrests the cell cycle in G_0_/G_1_ phase in lung cancer cells [20]; however, PES induces G_2_/M phase arrest in nasopharyngeal carcinoma [9]. In the present study, PES induced G_2_/M phase cell cycle arrest, induced apoptosis, and inhibited the growth of in OSCC cells *in vitro* and *in vivo*.

Clinical studies indicated that Hsp70 as a bad prognosis factor [23]. Increased expression of Hsp70 is associated with high-grade malignant tumors such as osteosarcoma, endometrial cancer, and renal cell tumors [23,24]. High expression of Hsp70 in gastric, endometrial, breast cancer, or leukemia has been reported to be relative with metastasis, poor prognosis, and resistance to chemotherapy or radiation therapy [25,26]. HSP70 acts as a ligand and binds to toll like receptor 4 (TLR4) thus activating the myeloid differentiation factor 88 (MyD88) pathway resulting in production of pro-inflammatory cytokines, chemokines, growth factors in OSCC [27]. XIAP and c-IAP1 are reported to be the clients of Hsp70, and interacted with Hsp70 [17,18]. In addition, XIAP and Hsp70 are found to be co-overexpressed in OSCC samples [28]. We found that PES inhibited expression of XIAP and c-IAP1 in OSCC cells. Importantly, PES disrupted the interaction between XIAP and Hsp70, which indicated that PES inhibited the biological function of Hsp70.

AKT and ERK signal transduction pathways play an important role in cell survival and regulation of apoptosis [29]. Previous studies have reported that PES decreases expression and phosphorylated level of AKT and ERK [9,20]. Our results were consistent with these studies. To response to the high level of stress condition, Hsp70 expresses abnormally high in cancer cells, and participates in oncogenesis and in resistance to chemotherapy [19]. Hence, various compounds are found to target Hsp70 and inhibit cancer growth. Quercetin and gemcitabine significantly enhanced apoptotic effect in lung cancer cells by inhibiting Hsp70 [30]. Triptolide decreased expression of Hsp70, which results in death of pancreatic cancer cell [31]. Down-regulated HSP70 with quercetin, OSCC cells displayed significant reduction in viability and an increase in apoptosis after plasmonic photothermal therapy [32]. Through overexpression and knockdown of Hsp70, our results suggested that PES inhibits growth of OSCC via Hsp70-denpendent manner.

## Conclusion

It is first suggested that PES, Hsp70 inhibitor, decreases cell viability in OSCC cells was revealed *in vivo* and *in vitro*. PES arrested cell cycle and induced apoptosis in OSCC cells. Notably, PES reduced the growth of OSCC cells by inhibiting the biological function of Hsp70.

## Abbreviations

CCLE: Cancer Cell Line Encyclopedia
c-IAP1: baculoviral IAP repeat containing 2
DMSO: dimethylsulfoxide
GAPDH: glyceraldehyde-3-phosphate dehydrogenase
Hsp: heat shock protein
Hsp70: heat shock protein 70
MyD88: myeloid differentiation factor 88
OSCC: oral squamous cell carcinoma
PES: 2-phenylethynesulfonamide
p-AKT: phosphorylated AKT
p-ERK: phosphorylated extracellular signal-regulated kinase
TLR4: toll like receptor 4
XIAP: X-linked inhibitor of apoptosis protein
